# Low percolation threshold of graphene/polymer composites prepared by solvothermal reduction of graphene oxide in the polymer solution

**DOI:** 10.1186/1556-276X-8-132

**Published:** 2013-03-22

**Authors:** Linxiang He, Sie Chin Tjong

**Affiliations:** 1 Department of Physics and Materials Science, City University of Hong Kong, Hong Kong, China; 2 Shenzhen Research Institute, City University of Hong Kong, Shenzhen, China

**Keywords:** Graphene, Composite, Percolation, Electrical conductivity

## Abstract

Graphene/polyvinylidene fluoride (PVDF) composites were prepared using *in-situ* solvothermal reduction of graphene oxide in the PVDF solution. The electrical conductivity of the composites was greatly improved by doping with graphene sheets. The percolation threshold of such composite was determined to be 0.31 vol.%, being much smaller than that of the composites prepared via blending reduced graphene sheets with polymer matrix. This is attributed to the large aspect ratio of the SRG sheets and their uniform dispersion in the polymer matrix. The dielectric constant of PVDF showed a marked increase from 7 to about 105 with only 0.5 vol.% loading of SRG content. Like the other conductor-insulator systems, the AC conductivity of the system also obeyed the universal dynamic response. In addition, the SRG/PVDF composite shows a much stronger nonlinear conduction behavior than carbon nanotube/nanofiber based polymer composite, owing to intense Zener tunneling between the SRG sheets. The strong electrical nonlinearity provides further support for a homogeneous dispersion of SRG sheets in the polymer matrix.

## Background

Recently, there is a great demand for polymeric materials with excellent electrical performance and high mechanical strength in the electronic industries. Conventional polymeric materials are insulators and can be made conductive by adding large volume fractions of conducting fillers in micrometer size such as metal and graphite particles [1-3]. However, high filler loadings generally result in low mechanical strength, heavy weight, and poor processability [4-6]. In this respect, fillers of nanometer dimensions are added to polymers to enhance their mechanical and physical performances [7-10]. Carbonaceous nanofillers such as carbon nanotubes (CNTs) with large mechanical strength and high electrical conductivity have been widely added to polymers to form conductive nanocomposites [11-17]. Their large aspect ratios enable the formation of conductive network in the polymer matrix at low filler contents. However, single-walled carbon nanotubes are very expensive, and the cost of multiwalled CNTs still remain relatively high despite a large reduction in their price in recent years [18]. The high cost of CNTs and their strong tendency to form aggregates have greatly limited their potential applications.

Graphite nanoplatelets (GNPs) prepared from the exfoliation of graphite intercalation compound (GIC) are low-cost fillers for preparing conductive polymer nanocomposites. The GIC can be synthesized by reacting natural graphite with electron-donor agents such as alkali metals or with electron acceptors [19]. However, GNPs consist of tens to hundreds of stacked graphene layers, corresponding to partially exfoliated graphite [20]. In 2004, Geim and co-workers successfully exfoliated graphite into graphene monolayer using the scotch tape method [21]. The monolayer graphene they obtained is believed to be a promising nanofiller for polymers due to its exceptionally high mechanical strength and excellent electrical and thermal properties. It has been reported that graphene/polymer composites exhibit much improved electrical and mechanical properties when compared to CNT/polymer composites [22,23]. In practice, however, the low yield of mechanically exfoliated graphene has greatly limited its applications. Thus, high-yield graphene oxide (GO) prepared from the chemical oxidation of graphite in strong oxidizing acids is commonly used to prepare graphene [24,25]. GO is electrically insulating; therefore, chemical reduction or thermal treatment is needed to restore its electrical conductivity [26,27]. In addition, graphene sheets have a great tendency to aggregate when they are loaded to the polymers. The aggregation is mainly due to the van der Waals attractions between the graphene sheets. This would deteriorate the electrical performance of the resultant composites, and usually, more fillers need to be loaded to form a percolating network in this case. In this study, we fabricated graphene/polymer composites using solvothermal reduction of GO in the polymer solution. This method enables the reduction of GO to graphene and its blending with the polymer matrix in one step. The polymer material used was polyvinylidene fluoride (PVDF). It is a semicrystalline polymer having remarkable thermal stability, excellent chemical resistance, and extraordinary pyro- and piezoelectric characteristics. It has found wide applications in the fields of electronic and biomedical engineering [28]. This study presents the first report on the synthesis and electrical characterization of the solvothermal reduced graphene/PVDF nanocomposites.

## Methods

### Materials

Graphite flakes and PVDF (Kynar 500) were purchased from Sigma-Aldrich Inc. (St. Louis, MO, USA) and Arkema Inc. (King of Prussia, PA, USA), respectively.

### Synthesis

Graphite oxide was prepared using a typical Hummers method [29]. In a typical composite fabrication procedure, graphite oxide was firstly ultrasonicated in *N, N*-dimethylformamide (DMF) for 40 min to be exfoliated into GO. PVDF pellets were then dissolved in this suspension at 60°C. Subsequently, the solution mixture was transferred into a 50-ml steel autoclave and placed in an oven at 100°C for 12 h. In this solvothermal reaction, DMF acted as the solvent for dissolving PVDF and also served as a medium to transmit heat and pressure to reduce GO. After the reaction ended, the autoclave was taken out and allowed to cool naturally, and a solution mixture of solvothermal reduced graphene (SRG) sheets and PVDF was obtained. This solution was used to fabricate the SRG/PVDF composites via the coagulation method [30]. In this process, the suspension was dropped into a blender containing a large amount of distilled water. The SRG/PVDF composite mixture precipitated out immediately due to its insolubility in the DMF/water mixture. The obtained fibrous SRG/PVDF mixture was vacuum filtrated and dried and finally hot-pressed into thin sheets of approximately 1 mm thick.

### Characterization

To convert wt.% loading of graphene sheets in the composite samples to vol.% (as used in the text), a density for the GO sheets of 2.2 g/cm^3^ was assumed [23]. The prepared GO was examined using an atomic force microscope (AFM, Veeco Nanoscope V, Plainview, NY, USA). The morphology of the SRG/PVDF composites was examined using a scanning electron microscope (SEM, Jeol JSM 820, JEOL Ltd., Akishima-shi, Japan). The dielectric constant and electrical conductivity of the composites were measured with a Hewlett Packard 4284A Precision LCR Meter (Hewlett-Packard Company, Palo Alto, CA, USA). The current density-electric field (*J*-*E*) characteristic of the composites was measured by a Hewlett Packard 4140B pA meter/DC voltage source (Hewlett-Packard Company, Palo Alto, CA, USA). Silver paste was coated on the specimen surfaces to form electrodes.

## Results and discussion

**Figure 1:**
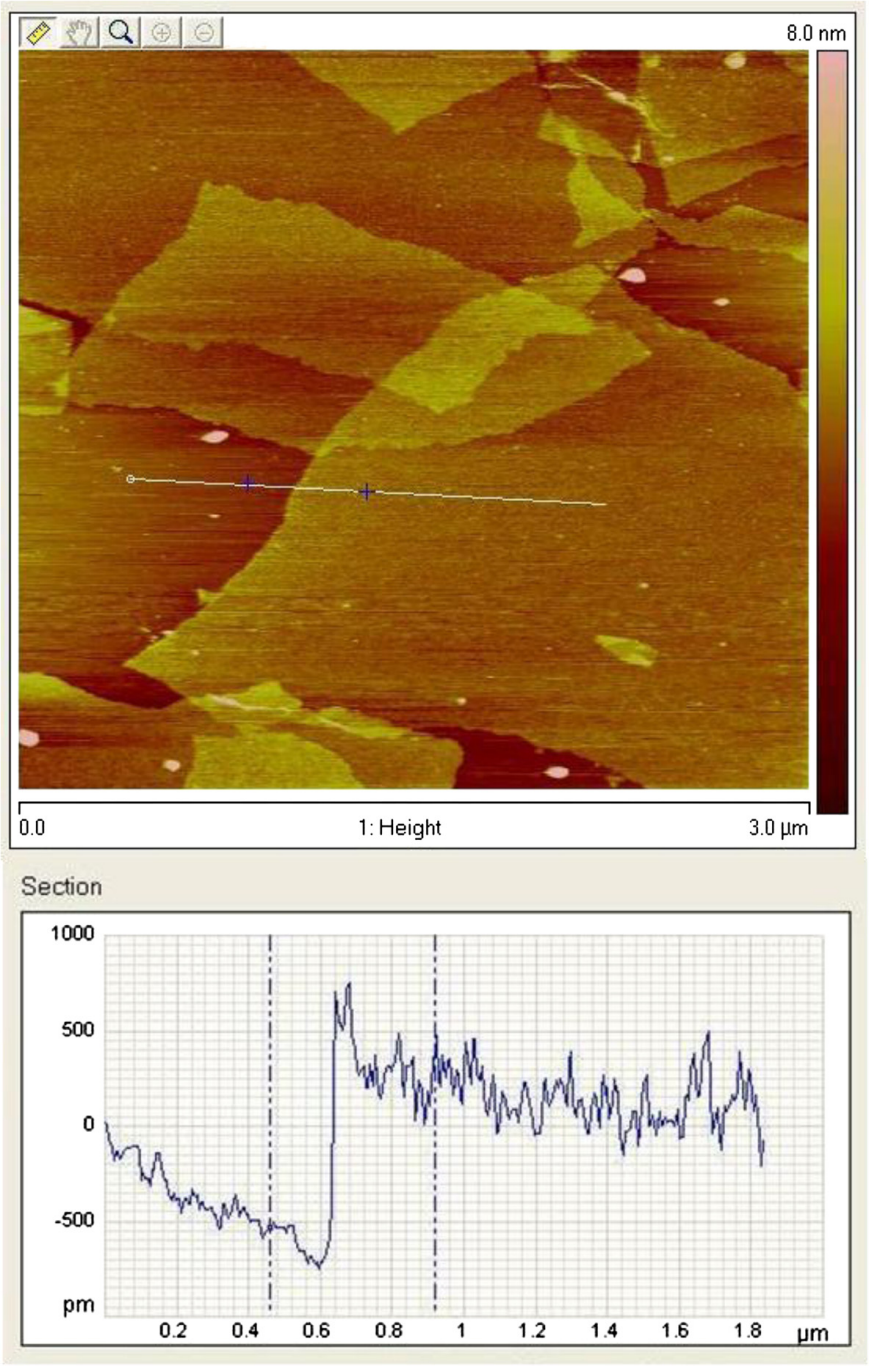
**AFM image of GO sheets on freshly cleaved mica.** The relative thickness across the horizontal line is approximately 1 nm, indicating the effective exfoliation of graphite oxide into monolayer GO sheets.

**Figure 2:**
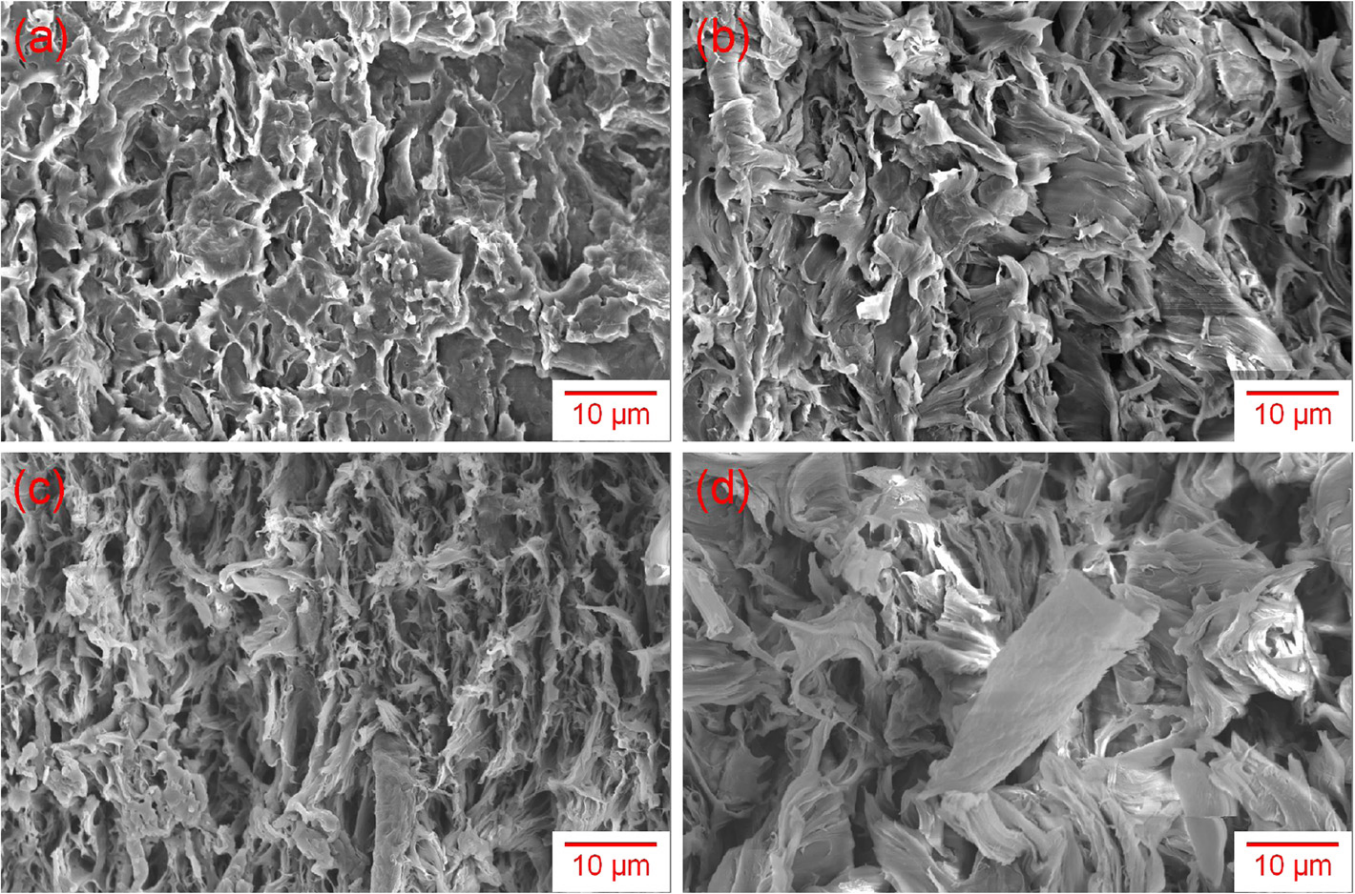
**SEM micrographs of PVDF nanocomposites.** (**a**) 0.4, (**b**) 0.5, (**c**) 0.8, and (**d**) 1.4 vol.% SRG sheets.

Figure 1 shows the AFM image of GO sheets prepared from chemical oxidation of graphite in strong acids. From the thickness measurements, it is obvious that the graphite oxide was completely exfoliated into monolayer GO. Because of the presence of carbonyl and carboxyl functional groups on its surface, the thickness of the sheets was approximately 1 nm, slightly thicker than graphene [31]. The average size of GO sheets was in the order of several micrometers, rendering them with very large aspect ratios. Figure 2 shows the morphology of SRG/PVDF composites containing different SRG loading levels. At low filler loadings, it is rather difficult to distinguish SRG sheets from the polymer matrix, due to its low contrast to the background and monolayer nature. As the filler content increases, the SRG sheets become more distinguishable, particularly at a filler content of 1.4 vol.%.

**Figure 3:**
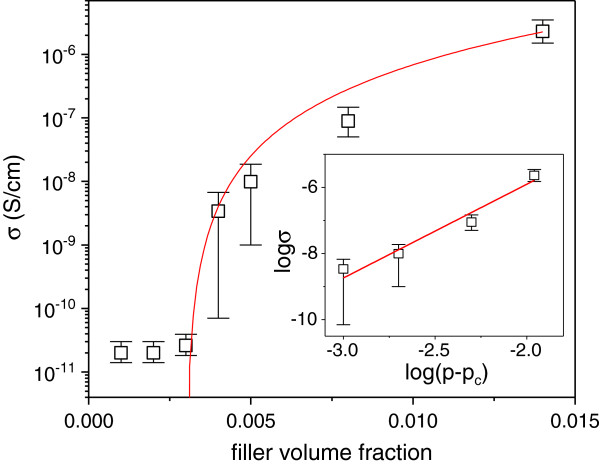
**Static conductivity of the SRG/PVDF composites showing percolative behavior.** The red solid lines are nonlinear fits to Equation 1. The conductivity takes the average value of ten samples. Inset is the plot of log *σ* versus log(*p*−*p*_c_).

The percolation theory is often employed to characterize the insulator-conductor transition of the polymer composites containing conductive fillers. Figure 3 shows the electrical conductivity versus filler content for the SRG/PVDF composites. According to the percolation theory, the static conductivity of the composites is given by [32,33]:

(1) $$\sigma \left(p\right)=\sigma_{0}{\left(p–p_{\text{c}}\right)}^{t},\mathrm{for}\;p>p_{\text{c}}$$

where *p*_c_ is the percolation threshold, *p* is the filler content, and *t* is the critical exponent. As shown in Table 1, the fit of electrical conductivity to Equation 1 yields a percolation threshold as low as 0.31 vol.% (Figure 3). Such a percolation threshold is lower than that of the graphene/PVDF composite prepared by direct blending chemically/thermally reduced GO sheets with polymers [34,35]. The low *p*_c_ is attributed to the homogeneous dispersion of SRG sheets within the PVDF matrix. In this study, we found that the SRG sheets could remain stable in the PVDF solution up to several weeks. Without PVDF in DMF, however, black SRG precipitates appeared after 1 day. So it is considered that the PVDF molecular chains could stabilize the SRG sheets. Since the GO sheets were enclosed by the PVDF molecular chains and reduced to SRG sheets during the solvothermal process, they would not fold easily or form aggregates as often happened. This would facilitate the formation of conducting network and result in a low percolation threshold. The large aspect ratios of the SRG sheets make the percolation threshold even smaller.

**Table 1:** Parameters characterizing percolative behavior of SRG/PVDF composites

**Composite**	***σ***_**0 **_**(S/cm)**	***p***_**c**_	***t *value**
SRG/PVDF	0.33	0.31 vol.%	2.64

**Figure 4:**
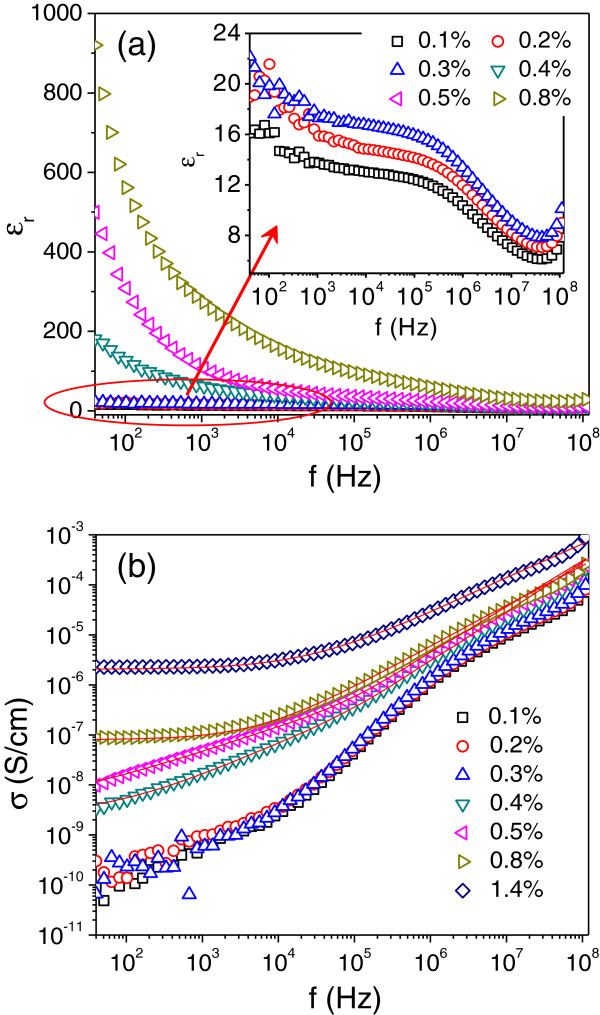
**Frequency dependency of (a) dielectric constant and (b) electrical conductivity of SRG/PVDF composite with various filler contents.** Inset in (**a**) shows dielectric constant versus frequency plots for the composites with 0.1, 0.2, and 0.3 vol.% SRG.

Figure 4a shows the frequency dependency of the dielectric constant (*ε*_r_) of the SRG/PVDF composites. Apparently, *ε*_r_ increases markedly with increasing SRG content. The increased *ε*_r_ can be attributed to the formation of various nanocapacitors consisting of SRG sheets separated by dielectric PVDF film [36-38]. At 1 kHz, the dielectric constant of pure PVDF is 7. This value reaches 60 and 105 when the PVDF was filled with 0.4 and 0.5 vol.% SRG, respectively. Although carbon-based polymeric composites with high dielectric permittivity have been reported [35,39-41], the dielectric loss of those composites are generally too large for practical applications. In contrast, the electrical conductivity of the SRG/PVDF composite (for *p* = 0.4 or 0.5 vol.%) is relatively low (see Figure 4b); therefore, the dielectric loss can be minimized. The good dielectric performance in combination with high flexibility makes such SRG/PVDF composite an excellent candidate of high-*k* material.

Figure 4b shows the variation of conductivity with frequency for SRG/PVDF composites. For the composites with low SRG loadings (*p* ≤ 0.3 vol.%), *σ*(*f*) increases almost linearly with frequency, which is a typical characteristic of insulating materials. When the filler content reaches 0.4 vol.% and above, *σ*(*f*) at low-frequency region shows a marked increase, due to the onset of the formation of percolating structure spanning the polymer matrix. For the composites with higher SRG loadings (*p* ≥ 0.8 vol.%), the conductivity is independent of the frequency at low-frequency regime. Above a characteristic frequency, the conductivity increases with increasing frequency. This indicates that a percolating SRG network throughout the whole system has been fully developed. The frequency-independent plateau is termed as the DC conductivity (*σ*_DC_) and particularly obvious for the composites with high SRG loadings. The two-stage conductivity behavior can be described by the following relationship [42,43]:

(2) $$\sigma \left(f\right)=\sigma \left(0\right)+\sigma \left(f\right)=\sigma_{\mathrm{DC}}+Af^{x}$$

where *A* is a constant depending on temperature and *x* is a critical exponent depending on both frequency and temperature. This behavior is typical for a wide number of conducting composite materials [42] and usually termed as ‘universal dynamic response’ [43,44]. Ezquerra et al. have had a detailed study of such a behavior [45-47]. We have also investigated this dynamic response in carbon nanotube/nanofiber based composites [48,49]. By fitting the data in Figure 4b to Equation 2, the values of *σ*_DC_, *A,* and *x* for percolative SRG/PVDF composites could be extracted. They are listed in Table 2.

**Table 2:** AC electrical transport properties of percolated SRG/PVDF composites

**Filler content**	***A***	***B***	***n *value**
0.4 vol.%	2.43×10^−9^ ± 2.12×10^−10^	1.42×10^−11^ ± 7.14×10^−12^	0.88 ± 0.01
0.5 vol.%	3.40×10^−9^ ± 8.13×10^−10^	3.23×10^−11^ ± 8.04×10^−12^	0.86 ± 0.01
0.8 vol.%	8.02×10^−8^ ± 1.34×10^−8^	6.14×10^−11^ ± 3.95×10^−12^	0.83 ± 0.01
1.4 vol.%	2.05×10^−6^ ± 7.90×10^−8^	1.44×10^−9^ ± 8.19×10^−11^	0.71 ± 0.01

**Figure 5:**
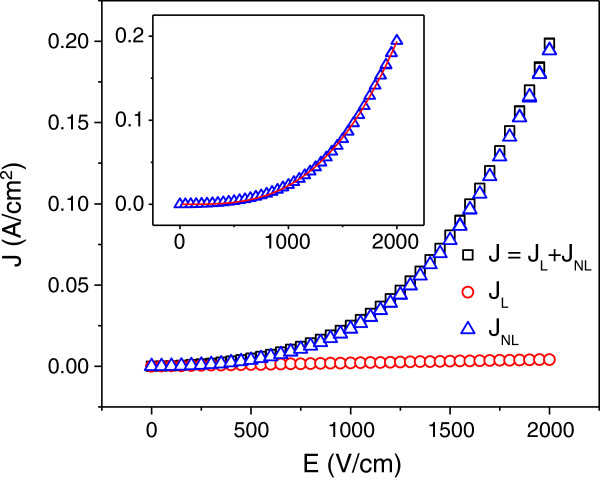
***J*-*E *characteristic of SRG/PVDF composite with *p *= 1.4 vol.%.** The inset shows the agreement of nonlinear current density (*J*_NL_) with Zener tunneling density *J* = *AE*^*n*^exp(−*B*/*E*).

Figure 5 presents the *J*-*E* characteristic of the PVDF composite with 1.4 vol.% SRG sheets. The composite exhibits a much stronger nonlinear conduction behavior compared with the polymer composites with carbon nanotubes/nanofibers [50]. Similarly, other SRG/PVDF composites with SRG content above *p*_c_ also exhibit such a behavior. As with other carbon/polymer composites, the current density *J* can be divided into linear *J*_*L*_ and nonlinear *J*_*NL*_. The nonlinear part is caused by the Zener tunneling of electrons between the SRG sheets. As shown in the inset of Figure 5, the Zener tunneling predicts the nonlinear current density (*J*_NL_) very well on the basis of the tunneling equation, i.e., *J* = *AE*^*n*^exp(−*B*/*E*) where *A*, *B*, and *n* are constants [51]. To the best of our knowledge, this is the first report about Zener effect in graphene/polymer composite. From our previous study, a homogeneous dispersion of conductive filler within the insulating matrix tends to cause strong Zener current [52]. Hence, the strong electrical nonlinearity provides further support for the uniform dispersion of the SRG sheets in the PVDF matrix.

## Conclusions

SRG/PVDF composite was prepared by *in-situ* solvothermal reduction of graphene oxide in the PVDF solution. The large aspect ratio of SRG sheets in combination with uniform dispersion in the polymer matrix led to a relatively low percolation threshold of 0.31 vol.%, which is smaller than graphene/polymer composites prepared by direct blending chemically/thermally reduced GO sheets with PVDF. It is found that only 0.5 vol.% SRG doping will increase the dielectric constant of the material from 7 to about 105, while keeping the conductivity at a low level. Such a dielectric performance is superior to those of carbon nanotube/nanofiber based polymeric composites. The AC conductivity of the composite above *p*_c_ follows the universal dynamic response, as with many other conductor-insulator systems. Moreover, the electrical nonlinearity of these composites is stronger than the carbon nanotube/nanofiber filled polymer system, resulting from the Zener tunneling effect between the uniformly dispersed SRG sheets.

## Competing interests

The authors declare that they have no competing interests.

## Authors’ contributions

LXH carried out the experiments, interpreted the data, and drafted the manuscript. SCT participated in the design of the study, material analysis, and revision of the whole manuscript. Both authors read and approved the final manuscript.
